# Ethical Issues Among Healthcare Workers During Covid-19 Pandemic

**DOI:** 10.1192/j.eurpsy.2026.11023

**Published:** 2026-07-31

**Authors:** N. Chaouch, H. Ziedi, S. Bey, G. Amri, R. Ghachem, N. Mechergui

**Affiliations:** 1 Occupational Medicine, Habib Thameur Hospital, Tunis; 2 B Department, Razi Hospital, Manouba, Tunisia

## Abstract

**Introduction:**

Ethical issues in the management of the coronavirus crisis are multiple, restrictions of freedoms by the imposition of containment, mandatory reporting of the disease affecting professional secrecy. Health care workers (HCWs) are on the front line in the management of this health crisis

**Objectives:**

Determine the acceptance of the compulsory declaration of the disease, the feeling of guilt and the rate of social and professional stigmatization by the care personnel.

**Methods:**

A descriptive cross-sectional study that interested HCWs at Charles Nicolle Hospital infected with SARS-COV2 who completed the return-to-work visit at the occupational medicine department, during the period from September 1 to December 31, 2020

**Results:**

During the study period 300 HCWs were included in the study. It was predominantly female with a sex ratio of 0.4. The mean age was 42±10 years. The HCWs were married in 70 % of cases. The professional categories were represented mainly by nurses in 36% of cases followed by technicians and physicians in 27% and 17.4% of cases respectively. The average professional seniority was 15 years (min=1 year; max=15 years).

Guilt was felt by 58% of the cases. Mandatory reporting of the disease was accepted by 98%.

Social stigma was reported by 32% and professional stigma by 21%. The stigmatized population was predominantly female (87%), the proportion of married people was 65%. The predominant professional category was nurses (36%) followed by senior technicians and workers (27% and 17% respectively). The average length of employment was 13 years.

**Conclusions:**

Based on the results of this study, the social and occupational stigma of the SARS-COV2-infected HCWs was significant as well as the feeling of guilt. Other ethical issues raised by this pandemic need to be studied in order to develop prevention measures.

**Disclosure of Interest:**

None Declared

